# Facial muscle movements in patients with Parkinson's disease undergoing phonation tests

**DOI:** 10.3389/fneur.2022.1018362

**Published:** 2022-10-31

**Authors:** Fan Xu, Xian-wei Zou, Li-qiong Yang, Shi-cong Mo, Quan-hao Guo, Jing Zhang, Xiechuan Weng, Guo-gang Xing

**Affiliations:** ^1^Department of Public Health, Chengdu Medical College, Sichuan, China; ^2^Department of Neurology, First Affiliated Hospital of Chengdu Medical College, Sichuan, China; ^3^School of Pharmacy, Chengdu Medical College, Sichuan, China; ^4^Department of Civil and Environment Engineering, Hong Kong Polytechnic University, Kowloon, Hong Kong SAR, China; ^5^MOEMIL Laboratory, Optoelectronic Science and Engineering, University of Electronic Science and Technology of China, Sichuan, China; ^6^Department of Neuroscience, Beijing Institute of Basic Medical Sciences, Beijing, China; ^7^Neuroscience Research Institute, Peking University, Beijing, China; ^8^Department of Neurobiology, School of Basic Medical Sciences, Peking University Health Science Center, Beijing, China; ^9^Key Laboratory for Neuroscience, Ministry of Education and Ministry of Health, Beijing, China

**Keywords:** Parkinson's disease, facial dystonia, phonation tests, facial expression, stiff muscle

## Abstract

**Purpose:**

Parkinson's disease (PD) is a serious neurodegenerative disease affecting the elderly. In general, the locomotion deficit, which seriously affects the daily life of patients with PD, usually occurs at a later stage. The mask face symptom meanwhile progressively worsens. However, facial muscle disorders and changes involved in the freezing mask are unclear.

**Method:**

In this study, we recruited 35 patients with PD and 26 age- and sex-balanced controls to undergo phonation tests, while the built-in camera on the laptop recorded their facial expressions during the whole pronunciation process. Furthermore, FaceReader (version 7.0; Noldus Information Technology, Wageningen, Netherlands) was used to analyze changes in PD facial landmark movement and region movement.

**Results:**

The two-tailed Student's *t*-test showed that the changes in facial landmark movement among 49 landmarks were significantly lower in patients with PD than in the control group (*P* < 0.05). The data on facial region movement revealed that the eyes and upper lip of patients with PD differed significantly from those in the control group.

**Conclusion:**

Patients with PD had defects in facial landmark movement and regional movement when producing a single syllable, double syllable, and multiple syllables, which may be related to reduced facial expressions in patients with PD.

## Introduction

Parkinson's disease (PD) is a common neurological degenerative disease. From 1990 to 2016, the standard incidence ratio of PD increased by 21.7% (1). The cost of patients with PD in China exceeds the average economic capacity, especially anti-Parkinson medication and caring costs (2).

Decreased facial movement is a clinical feature of PD, known as “masked syndrome.” Approximately 39–65% of patients with PD experience freezing mask (3). Freezing mask is associated with dysarthria and dysphonia. Dysarthria is caused by neurologic damage to the motor components of speech, which may involve any or all of the speech processes, including respiration, phonation, articulation, resonance, and prosody. Our previous studies demonstrated that dysarthria was primarily manifested by sound quality changes, poor clarity, decreased volume, trembling, and hoarseness (4). In addition, dysphonia refers to disordered sound production at the level of the larynx, classically seen as hoarseness. It may have a neurologic, structural, or functional etiology (5). Fluorographic studies have demonstrated that the most common progression of vocal tract symptoms begins with laryngeal dysfunction, followed by changes in tongue and lip function (6). Stiffness of the laryngeal muscle tissue usually increases the hardness of the vocal cords, thereby affecting the closure of the vocal cords and increasing muscle tone (7). In addition, freezing mask may occur when muscle stiffness extends to the face. The main manifestation of mask face is the movement of the eyebrows, eyes, cheeks, and lips, and other movements have serious obstacles in speed, elasticity, and coordination. The facial muscles consequently become increasingly stiff (8, 9), which is caused by the inhibition of muscle activity responsible for facial expressions (10).

The face contains 44 muscles. The interaction of these organizations creates abundant facial expressions, including happiness, anger, despair, and other emotions (11). Happy and fearful faces activate the amygdala bilaterally, whereas sad faces only activate the right amygdala; disgust seems to preferentially activate the anterior insula (12) and fear preferentially activates the amygdala (13). A smile is accompanied by raised cheeks in the upper half of the face (14). Surprise is usually manifested as stretched eyebrows and an open mouth, whereas anger is manifested as an open mouth and frown (15).

These dominant facial expressions are accompanied by facial muscle activation (stretching or shrinking). However, facial muscle stiffness in patients with PD reduces these expressions. Numerous studies (16, 17) have validated the difficulty in identifying the expressions of sadness, anger, and fear in patients with PD. Gunnery et al. (18) measured spontaneous facial expressions across 600 frames in patients with PD and found that, if the severity of facial expression deficit was increased, the number, duration, intensity, and coactivation of facial muscle action was decreased. In addition, another study (19) demonstrated that, compared to healthy individuals, patients with PD had more difficulty identifying negative emotions (e.g., anger, disgust, fear, and sadness) than identifying relatively positive emotions (e.g., happiness, surprise). Accumulative evidence has shown that the ability of patients with PD to recognize aversive and neutral facial expressions in the early stages of the disease is significantly lower than that of the control group. Identifying other facial expressions (e.g., fear, sadness) is also weaker among patients with PD than among the control group (20, 21).

A freezing mask generally occurs early in the course of PD because of the loss of spontaneous facial expression and dystonic contraction of the facial muscles (10). Marneweck et al. (22) reported that facial muscle autonomic control was impaired in most patients with PD and was positively and highly correlated with disease severity (22). With regard to motor symptoms, patients experience hypomimia (e.g., spontaneous blinking and reduced facial expressions), which often occurs in the early phase of the disease. In stage IV PD, facial muscles become increasingly rigid; therefore, the richness of facial expressions is significantly decreased (23). In 2016, Livingstone et al. (24) found that the frontal muscles of patients with PD had a weakened response to a sad expression. In 2019, Okamoto et al. used FaceReader (Noldus Information Technology) and surface electromyography to conduct a three-dimensional facial expression analysis of patients with PD to evaluate facial expression and muscle activity, respectively. Patients with PD in the intervention group were treated with facial rehabilitation exercises. Patients with PD had a lower “happy” index and a higher “sad” index. Facial rehabilitation exercises affected the emotions, facial expressions, and facial muscle activity of patients with PD (25).

Therefore, facial expression loss in patients with PD often manifests before motor symptoms and occurs in the early stage. However, little is known about how coordinated movements across regions of the face are impaired in PD. Furthermore, at present, the micro-stiffness of the facial muscles in the freezing mask is difficult to recognize—that is, no software can recognize facial muscle movements more sensitively. In this study, we used facial landmarks to identify the micro-movement of each facial muscle during the phonation test to explore facial region movement.

## Materials and methods

### Ethics statement

The Institute of Institutional Review Board and Ethics Committee of the First Affiliated Hospital of Chengdu Medical College (Sichuan, P.R. China) approved this study. Written informed consent was provided by all participants.

### Participants

From January to December 2019, two groups of participants were recruited: patients with PD and healthy individuals (i.e., the control group). The PD group consisted of 35 patients, including 21 men and 14 women, the average age was 67.57 ± 8.78 years. The control group consisted of 26 age- and sex-balanced healthy participants, which included 11 men and 15 women, and the average age was 66.46 ± 7.02 years. The severity of PD was evaluated, based on the Hoehn–Yahr Scale (H&Y) and the Unified Parkinson's Disease Rating Scale III (UPDRS III), and the duration of the disease was recorded. In addition, profession, alcohol consumption, smoking habits, and education level of all participants were recorded. The entire recording and evaluation process was conducted by a neurologist. All patients with PD included in the study met the following main inclusion criteria: (1) the patient's neurologist had undergone PD and other movement disorder management training and had diagnosed the patient as having idiopathic PD; and (2) during the first 3 months of the study, the patient had not participated in other clinical trials. The exclusion criteria for all participants were as follows: (1) a history of other neurological diseases; (2) severe mental disorders or cognitive disorders that may hinder speech; (3) mental illness or major systemic diseases; (4) clinical problems such as aphasia; (5) a medical history of acute stroke, sports injury, or mental illness; (6) failure to complete the learning task accurately; and (7) participation in other rehabilitation projects. Patients with PD stopped taking levodopa on the morning of the phonation test but continued to take other anti-Parkinson's drugs. All patients were in the “ON” stage. If a patient had severe motor symptoms, the experiment was not conducted.

### Phonation tests

Vowels have an important role in Chinese Pinyin. The pronunciation of vowels requires the tongue, lip, and jaw to form an oropharyngeal resonance cavity. When vowel sounds are produced, the airflow exhaled from the lungs passes through the mouth with minimal resistance and no friction sound (26). This process is suitable for studying patients with PD who have low oral pressure and dysphonia. In this study, we chose the vowels /a/, /o/, and /e/ to form the syllables “lā lā lā,” “duǒ,” and “fēi é,” respectively, for the phonation test. Furthermore, our previous research has verified its feasibility and accuracy (27).

### Face muscle movements recording and analysis

The participants were guided through playing the slides of “lā lā lā,” “duǒ,” and “fēi é” on a laptop. The built-in camera on the laptop recorded facial movement during the entire test process. FaceReader (version 7.0; Noldus Information Technology, Wageningen, Netherlands) was used to analyze detail parameters, including landmarks of key points on the face, head orientation, mouth, eye, and eyebrow open or closed status. Clinicians cross-validated and ensured quality control of the video recording. Ensuring that a participant's face had even lighting was important. The participant maintained normal intonation and loudness in a relaxed state. All participants were under the guidance of clinicians. If a participant felt tired, the test was suspended until the participant was satisfied with completing the rest of the test.

### Statistical analysis

All data were stored in Excel (Microsoft Corporation, Redmond, WA, USA). All analyses were conducted using STATA15.0 (Stata Corporation, College Station, TREATMENT, USA). The Chi-square test was used to compare the distribution of the participant's sex, profession, alcohol consumption, smoking habit, and education level between the two groups. The data were expressed as the mean ± standard deviation. The two-tailed Student's *t*-test was used to assess whether differences existed between the two groups in age, landmark movement, and facial region movement. A value of *P* < 0.05 was considered statistically significant.

## Results

### General information

In this study, the demographic characteristics of 35 patients with PD and 26 control individuals were compared. Table 1 displays no significant difference between the two groups in age (*t* = 0.5305, *df* = 59*, P* = 0.7011) and sex (χ^2^ = 1.874*, df* = 1, *P* = 0.171). In addition, the duration of PD was 4.59 ± 3.75 years, and the average scores on the H&Y and UPDRS III were 2.60 ± 0.81 and 35.60 ± 20.39, respectively. However, no significant difference existed in alcohol consumption and smoking habits between the two groups. The results revealed a significant difference in profession (χ^2^ = 6.2674, *df* = 2*, P* = 0.044) and education level (χ^2^ = 8.8961*, df* = 3, *P* = 0.03) between the two groups.

**Table 1:** Baseline characteristics of the participants.

**Variable**	**Patients with PD**	**Control group**	***P*-value**
Number	35	26	
Age, y*	67.57 ± 8.78	66.46 ± 7.02	*P =* 0.70
Sex, M/F^#^	21/14	11/15	*P =* 0.17
Duration of disease, y	4.59 ± 3.75	-	
H&Y Scale score	2.60 ± 0.81	-	
UPDRS III score	35.60 ± 20.39	-	
Alcohol consumption, N/Y^#^	27/8	23/3	*P =* 0.26
Smoker, N/Y^#^	26/9	23/3	*P =* 0.17
**Profession** ^**#**^			*P =* 0.04
Retired	10	1	
Farmer	17	18	
Worker	8	7	
**Education** ^**#**^			*P =* 0.03
Primary school	19	23	
Middle school	10	1	
High school	5	2	
Master's degree	1	0	

The data are presented as the number or as the mean ± standard deviation.

# Based on the Chi-square test.

* Based on the two-tailed Student's t-test.

PD, Parkinson's disease; M, male; F, female; H&Y, Hoehn–Yahr Scale; UPDRS III, Unified Parkinson's Disease Rating Scale III; N, no; Y, yes.

### Landmark movement

**Figure 1:**
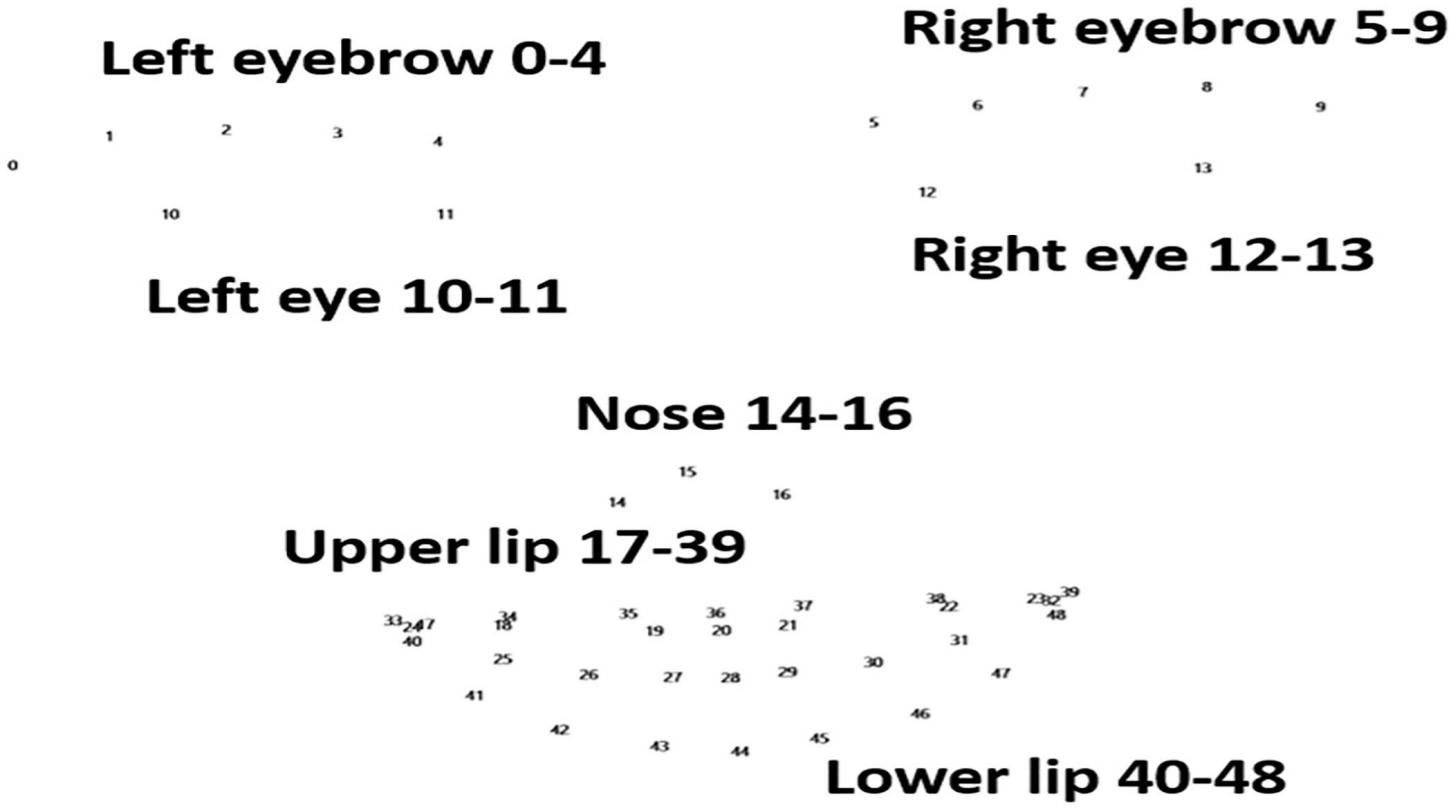
Landmarks of the face. Numbers 0–48 represent the 49 landmarks in the face, involving the eyebrows, eyes, nose, upper lip, and lower lip. These are the points recognized by FaceReader (version 7.0; Noldus Information Technology, Wageningen, Netherlands).

Changes in landmark movement between patients with PD and the controls were compared during the phonation test. Details of the landmarks on the face are listed in Figure 1. The data revealed that changes in 49 landmarks were significantly lower in patients with PD than in the controls (Table 2). This finding was consistent with the manifestation of stiff facial muscles in patients with PD. Interestingly, patients with PD had a significantly higher mean of the landmarks in “duǒ” and “lā lā lā.” This result contrasted with that of the controls, who had a significant decreased mean of landmarks in “duǒ” than “lā lā lā.” This finding indicated that different pronunciations may have different effects on facial muscle movements in patients with PD.

**Table 2:** Comparison of landmark movements in groups due to syllable pronunciation.

	**Pronunciation “lā lā lā”**			**Pronunciation “duǒ”**		
**Landmarks**	**Patients with PD**	**Control**	***P*-value**	**Patients with PD**	**Control**	***P*-value**
	**Mean ± S.Dev**	**Mean ± S.Dev**		**Mean ± S.Dev**	**Mean ± S.Dev**	
L0	0.0059 ± 0.0037	0.0231 ± 0.0285	0.0211*	0.0065 ± 0.0085	0.0227 ± 0.0300	0.0403*
L1	0.0058 ± 0.0040	0.0236 ± 0.0290	0.0194*	0.0064 ± 0.0077	0.0231 ± 0.0302	0.0344*
L2	0.0059 ± 0.0041	0.0242 ± 0.0295	0.018*	0.0063 ± 0.0072	0.0235 ± 0.0307	0.0316*
L3	0.0059 ± 0.0042	0.0247 ± 0.0299	0.0167*	0.0063 ± 0.0068	0.0238 ± 0.0311	0.0308*
L4	0.0057 ± 0.0042	0.0249 ± 0.0303	0.0164*	0.0062 ± 0.0069	0.0240 ± 0.0317	0.0309*
L5	0.0059 ± 0.0046	0.0257 ± 0.0315	0.0168*	0.0066 ± 0.0081	0.0251 ± 0.0338	0.0361*
L6	0.0061 ± 0.0047	0.0259 ± 0.0316	0.0172*	0.0069 ± 0.0084	0.0254 ± 0.0341	0.0381*
L7	0.0060 ± 0.0045	0.0258 ± 0.0315	0.017*	0.0068 ± 0.0085	0.0254 ± 0.0344	0.0385*
L8	0.0059 ± 0.0044	0.0256 ± 0.0315	0.0173*	0.0067 ± 0.0085	0.0254 ± 0.0347	0.0392*
L9	0.0058 ± 0.0041	0.0253 ± 0.0314	0.018*	0.0066 ± 0.0085	0.0254 ± 0.0351	0.0406*
L10	0.0056 ± 0.0037	0.0233 ± 0.0291	0.0199*	0.0063 ± 0.0087	0.0230 ± 0.0309	0.0394*
L11	0.0054 ± 0.0038	0.0239 ± 0.0300	0.0183*	0.0060 ± 0.0080	0.0236 ± 0.0321	0.0364*
L12	0.0055 ± 0.0041	0.0248 ± 0.0310	0.0174*	0.0061 ± 0.0077	0.0246 ± 0.0341	0.0374*
L13	0.0057 ± 0.0041	0.0252 ± 0.0314	0.0179*	0.0064 ± 0.0081	0.0253 ± 0.0349	0.0383*
L14	0.0055 ± 0.0044	0.0261 ± 0.0324	0.0156*	0.0063 ± 0.0078	0.0253 ± 0.0344	0.0343*
L15	0.0059 ± 0.0048	0.0271 ± 0.0329	0.0148*	0.0067 ± 0.0084	0.0260 ± 0.0346	0.0332*
L16	0.0055 ± 0.0045	0.0265 ± 0.0328	0.0154*	0.0063 ± 0.0082	0.0256 ± 0.0350	0.0353*
L17	0.0054 ± 0.0035	0.0251 ± 0.0317	0.0177*	0.0062 ± 0.0071	0.0247 ± 0.0349	0.0407*
L18	0.0055 ± 0.0038	0.0255 ± 0.0321	0.0178*	0.0063 ± 0.0072	0.0250 ± 0.0350	0.0394*
L19	0.0057 ± 0.0044	0.0262 ± 0.0328	0.017*	0.0064 ± 0.0077	0.0256 ± 0.0355	0.0374*
L20	0.0056 ± 0.0044	0.0263 ± 0.0329	0.0168*	0.0064 ± 0.0079	0.0257 ± 0.0356	0.0375*
L21	0.0056 ± 0.0043	0.0264 ± 0.0331	0.0164*	0.0064 ± 0.0081	0.0258 ± 0.0358	0.0377*
L22	0.0056 ± 0.0044	0.0263 ± 0.0331	0.0171*	0.0064 ± 0.0082	0.0259 ± 0.0364	0.0393*
L23	0.0057 ± 0.0045	0.02630 ± 0.033	0.0175*	0.0063 ± 0.0082	0.0259 ± 0.0366	0.0394*
L24	0.0055 ± 0.0036	0.0252 ± 0.0317	0.0175*	0.0062 ± 0.0071	0.0248 ± 0.0349	0.0402*
L25	0.0056 ± 0.0037	0.0260 ± 0.0320	0.0156*	0.0064 ± 0.0072	0.0253 ± 0.0352	0.0386*
L26	0.0058 ± 0.0039	0.0266 ± 0.0322	0.0146*	0.0065 ± 0.0073	0.0257 ± 0.0354	0.0371*
L27	0.0059 ± 0.0040	0.0269 ± 0.0325	0.0143*	0.0066 ± 0.0076	0.0260 ± 0.0357	0.0365*
L28	0.0059 ± 0.0041	0.0270 ± 0.0327	0.0142*	0.0066 ± 0.0077	0.0261 ± 0.0359	0.0365*
L29	0.0059 ± 0.0041	0.0271 ± 0.0328	0.0143*	0.0066 ± 0.0079	0.02620 ± 0.036	0.0366*
L30	0.0058 ± 0.0042	0.0271 ± 0.0329	0.0145*	0.0066 ± 0.0080	0.0262 ± 0.0362	0.0367*
L31	0.0057 ± 0.0043	0.02670 ± 0.033	0.0155*	0.0064 ± 0.0081	0.0261 ± 0.0364	0.0378*
L32	0.0057 ± 0.0045	0.0264 ± 0.0330	0.0167*	0.0063 ± 0.0082	0.0260 ± 0.0365	0.0382*
L33	0.0055 ± 0.0035	0.0251 ± 0.0316	0.0178*	0.0062 ± 0.0071	0.0247 ± 0.0348	0.0406*
L34	0.0057 ± 0.0040	0.0257 ± 0.0322	0.0176*	0.0064 ± 0.0073	0.02520 ± 0.035	0.0384*
L35	0.0057 ± 0.0044	0.0263 ± 0.0327	0.0167*	0.0065 ± 0.0077	0.0256 ± 0.0351	0.037*
L36	0.0058 ± 0.0045	0.02650 ± 0.033	0.0167*	0.0066 ± 0.0080	0.0258 ± 0.0354	0.0373*
L37	0.0058 ± 0.0045	0.0266 ± 0.0331	0.0167*	0.0066 ± 0.0083	0.0259 ± 0.0357	0.038*
L38	0.0056 ± 0.0044	0.0265 ± 0.0331	0.0164*	0.0064 ± 0.0083	0.0259 ± 0.0362	0.038*
L39	0.0057 ± 0.0045	0.02640 ± 0.033	0.0169*	0.0063 ± 0.0083	0.0260 ± 0.0366	0.0388*
L40	0.0055 ± 0.0036	0.0253 ± 0.0317	0.0174*	0.0063 ± 0.0071	0.0248 ± 0.0350	0.0402*
L41	0.0058 ± 0.0038	0.0262 ± 0.0323	0.0161*	0.0065 ± 0.0072	0.0255 ± 0.0355	0.039*
L42	0.0059 ± 0.0039	0.0268 ± 0.0325	0.0148*	0.0067 ± 0.0074	0.0260 ± 0.0358	0.0376*
L43	0.0061 ± 0.0041	0.0273 ± 0.0329	0.0144*	0.0068 ± 0.0077	0.0264 ± 0.0361	0.0369*
L44	0.0061 ± 0.0041	0.0275 ± 0.0332	0.0142*	0.0069 ± 0.0079	0.0266 ± 0.0364	0.037*
L45	0.0061 ± 0.0042	0.0277 ± 0.0333	0.014*	0.0069 ± 0.0081	0.0267 ± 0.0366	0.0368*
L46	0.0060 ± 0.0043	0.0275 ± 0.0334	0.0147*	0.0067 ± 0.0082	0.0267 ± 0.0368	0.0374*
L47	0.0058 ± 0.0044	0.0271 ± 0.0334	0.0157*	0.0066 ± 0.0083	0.0264 ± 0.0368	0.0382*
L48	0.0057 ± 0.0044	0.0265 ± 0.0331	0.0166*	0.0063 ± 0.0083	0.0261 ± 0.0366	0.0384*

L0 to L48 indicates Landmark 0 to Landmark 48.

* A value of P < 0.05 is significant.

PD, Parkinson's disease; S.Dev, standard deviation.

### Facial region movement

We also compared facial region movement between the two groups. Both eyes and the upper lip were interestingly significantly different between the two groups. In particular, when pronouncing “lā lā lā,” the average movement of the left and right eyes of patients with PD was significantly lower than that of the controls (*P* < 0.05). The average movement of the right eye with the syllable “duǒ” was similarly significantly lower in patients with PD than in the controls (*P* < 0.05). This finding may be related to the fact that patients with PD blink less frequently than healthy individuals. However, when patients with PD pronounced “fēi é,” their upper lip movement was significantly higher than that of the control group (*P* < 0.05), (see Table 3).

**Table 3:** Comparison of face regions in groups, based on syllable pronunciation.

**Face region**	**Pronunciation**	**Patients with PD** **(mean ±S.Dev)**	**Control** **(mean ±S.Dev)**	***P-*value**
Left eye	lā lā lā	0.0829 ± 0.0468*	0.1421 ± 0.0996*	0.0329
Right eye	lā lā lā	0.0778 ± 0.0310*	0.1321 ± 0.0879*	0.0227
	duǒ	0.0604 ± 0.0306*	0.1214 ± 0.1018*	0.0246
Upper lip	fēi é	0.0459 ± 0.0186*	0.0322 ± 0.0181*	0.0373

* A value of P < 0.05 is significant.

S.Dev, standard deviation; PD, Parkinson's disease.

## Discussion

One of the most common movement symptoms of PD is a facial muscle movement disorder, including the reduction and slowing of facial muscle movement, which may affect the upper and lower parts of the face: in the upper part, it is shown that the blink rate and blink range decreases. In the lower part, the displacement amplitude of the jaw and upper lip decreases (9, 10). In addition, facial muscle movement disorder makes PD patients to have serious problems such as speech disorder, dysphagia, and salivation (10, 28). In this study, the movement of the 49 landmarks in patients with PD was significantly decreased, compared to the movements in the control group. This finding indicated that the movements of the left and right eyebrows, left and right eyes, nose, and upper and lower lips of patients with PD were weakened. This finding was consistent with the symptoms of facial freezing in patients with PD. The freezing mask is usually manifested by the reduction of the voluntary movement of the facial muscle group and the obvious reduction of the movement range, so that the facial expression becomes not obvious, resulting in facial expression disorder. This is mainly caused by damage to the nervous system of speech motor components.

Both groups had significant differences in the left eye, right eye, and upper lip movements when they pronounced the same syllable. During the phonation test, patients with PD in our present study had smaller eye muscle movements than did those in the control group. This factor may be the reason why PD patients usually have mild to obvious facial expression reduction and spontaneous blinks/minute, which usually represents an early motor symptom of PD (29).

Furthermore, a lack of movement in certain parts of the patient's body, including the face, neck, and arms, can also be an early sign of the disease. PD is a neurodegenerative disease caused by the loss of dopaminergic neurons in the dense part of the substantia nigra of the brain (30). The loss of these neurons leads to the reduction of dopamine neurotransmission in the basal ganglia, resulting in excessive inhibition of some facial movements and cognitive pathways (31). When PD damages dopamine-producing nerve cells, the ability of nerves to control muscles is affected, resulting in the appearance of facial movement symptoms (32). Basal ganglia dysfunction associated with PD may contribute to orofacial movement disorders. An abnormal increase in driving neuromotor activity mediated by lower motor neuron centers translates to an increase in muscle stiffness, which is the clinical correlate of muscular rigidity in PD. The main manifestations of freezing mask are serious obstacles in the speed, elasticity, and coordination of the eyebrows, eyes, cheeks, lips, and so on (9).

Taken together, patients with PD presented significantly different facial muscle movements during the phonation test. The pronunciation of different syllables requires a different coordination mechanism, such as the breath muscle movement-induced vocal cord vibration. Because the spontaneous and non-spontaneous movements of the face depend on the fine coordination between the complex facial nervous and muscular systems. According to the observation of facial muscle movement, it is a useful attempt to diagnose and monitor PD patients at an early stage. Therefore, these valuable evidence are helpful for the early diagnosis and monitoring of PD.

### Limitations

The patients with PD in this study stopped taking levodopa before the experiment but continued to take other anti-Parkinson's disease drugs. Thus, the patients were in the “ON” stage. In addition, this experiment did not conduct a facial electromyographic study. Furthermore, the larger sample size in further studies could be the strength of the conclusion, and consequently, consider the interesting findings as preliminary.

## Data availability statement

The original contributions presented in the study are included in the article/supplementary material, further inquiries can be directed to the corresponding author/s.

## Ethics statement

The studies involving human participants were reviewed and approved by the Ethics Committee of Chengdu Medical College. The patients/participants provided their written informed consent to participate in this study.

## Author contributions

FX, L-qY, and G-gX developed the study concept and drafted the manuscript with X-wZ. X-wZ and Q-hG recruited the subjects and collected the phonation test and facial expression data. JZ performed the Noldus FaceReader 7.0 operation, and conducted the statistical analysis with Q-hG. XW and S-cM proofread the manuscript. All authors contributed to the article and approved the submitted version.

## Funding

This work was supported by the Chengdu Medical College Natural Science Foundation (CYZ18-33, CYZ19-33), the Sichuan Provincial Education Department (17ZA0134), the Sichuan Medical Association (S18023), Chengdu Science and Technology Bureau focuses on research and development support plan (2019-YF09-00097-SN), the popular scientific research project of Sichuan Health Commission (20PJ171), and Sichuan undergraduate innovation and startup program funding support (S201913705080, S201913705130, S201913705059, S202013705070, S202013705075, S202013705108).

## Conflict of interest

The authors declare that the research was conducted in the absence of any commercial or financial relationships that could be construed as a potential conflict of interest.

## Publisher's note

All claims expressed in this article are solely those of the authors and do not necessarily represent those of their affiliated organizations, or those of the publisher, the editors and the reviewers. Any product that may be evaluated in this article, or claim that may be made by its manufacturer, is not guaranteed or endorsed by the publisher.

## Abbreviations

PD: Parkinson's disease.
